# Supplementary material for: New Polyketide and Butenolide Derivatives from the Mangrove Fungus Aspergillus spelaeus SCSIO 41433
Source: Mar Drugs. 2025 Jun 13;23(6):251. doi: 10.3390/md23060251 (PMC12194153; doi:10.3390/md23060251)
Supplement: Supplementary file 1 [file marinedrugs-23-00251-s001.zip › marinedrugs-3685277-supplementary.pdf]

Supporting Information

New Polyketide and Butenolide Derivatives from the Mangrove Fungus *Aspergillus spelaeus* SCSIO 41433

Zimin Xiao [1,†], Jiaqi Liang [2,3,†], Chun Yang [1], Jian Cai [2,3], Bin Yang [2], Xuefeng Zhou [2], Jie Yuan [4,*] and Huaming Tao [1,*]

[1] School of Traditional Chinese Medicine, Southern Medical University, Guangzhou 510515, China; 15917491112@163.com (Z.X.); 3201008013@smu.edu.cn (C.Y.)
[2] Guangdong Key Laboratory of Marine Materia Medica/State Key Laboratory of Tropical Oceanography, South China Sea Institute of Oceanology, Chinese Academy of Sciences, Guangzhou 510301, China; liangjiaqi22@mails.ucas.ac.cn (J.L.); caijian@scsio.ac.cn (J.C.); yangbin@scsio.ac.cn (B.Y.); xfzhou@scsio.ac.cn (X.Z.)
[3] University of Chinese Academy of Sciences, Beijing 100049, China
[4] Zhongshan School of Medicine, Sun Yat-sen University, Guangzhou 510080, China
* Correspondence: yuanjie@mail.sysu.edu.cn (J.Y.); taohm@smu.edu.cn(H.T.)
† These authors contributed equally to this work.

Table of Contents

The physicochemical data of the known compounds **3-22**.

Figure S1. ^{1}H NMR spectrum of (±)-penilactone F (**1**) in $CDCl_3$

Figure S2. ^{13}C NMR spectrum of (±)-penilactone F (**1**) in $CDCl_3$

Figure S3. HSQC spectrum of (±)-penilactone F (**1**) in CD_3OD

Figure S4. HMBC spectrum of (±)-penilactone F (**1**) in CD_3OD

Figure S5. ^{1}H-^{1}H COSY spectrum of (±)-penilactone F (**1**) in $CDCl_3$

Figure S6. HRESIMS spectrum of (±)-penilactone F (**1**)

Figure S7. IR spectrum of (±)-penilactone F (**1**)

Figure S8. UV spectrum of (±)-penilactone F (**1**) in CH_3OH

Figure S9. ECD spectrum of (±)-penilactone F (**1**) in CH_3OH

Figure S10. ECD spectrum of (+)-penilactone F ((+) -**1**) in MeOH

Figure S11. ECD spectrum of (−)-penilactone F ((-) -**1**) in MeOH

Figure S12. ^{1}H NMR spectrum of (±) phenylbutyrolactone IIa (**2**) in CD_3OD

Figure S13. ^{13}C NMR spectrum of (±) phenylbutyrolactone IIa (**2**) in CD_3OD

Figure S14. DEPT135 spectrum of (±) phenylbutyrolactone IIa (**2**) in CD_3OD

Figure S15. HSQC spectrum of (±) phenylbutyrolactone IIa (**2**) in CD_3OD

Figure S16. HMBC spectrum of (±) phenylbutyrolactone IIa (**2**) in CD_3OD

Figure S17. HRESIMS spectrum of (±) phenylbutyrolactone IIa (**2**)

Figure S18. IR spectrum of (±) phenylbutyrolactone IIa (**2**)

Figure S19. UV spectrum of (±) phenylbutyrolactone IIa (**2**) in CH_3OH

Figure S20. ECD spectrum of (±) phenylbutyrolactone IIa (**2**) in CH_3OH

The strain's (*Aspergillus spelaeus* SCSIO 41433) ITS sequence of the rDNA

Compound **3**: Pale yellow powder; ^{1}H NMR (CD_3OD, 600 MHz) δ 7.63 (2H, m, H-2″, H-6″), 7.28 (2H, dd, J = 8.4, 7.0 Hz, H-3″, H-5″), 7.23 (1H, m, H-4″), 7.16 (2H, m, H-2′, H-6′), 6.73 (2H, m, H-3′, H-5′), 6.25 (1H, d, J = 1.4 Hz, H-4); ^{13}C NMR (CD_3OD, 150 MHz) δ 171.5 (C-1), 159.7 (C-4′), 140.2 (C-2), 132.2 (C-3), 130.6 (C-2′, C-6′), 129.5 (C-1″), 129.4 (C-4″), 129.3 (C-3″, C-5″), 128.9 (C-2″, C-6″), 128.4 (C-1′), 116.7 (C-3′, C-5′), 82.4 (C-4).

Compound **4**: Brown powder; ^{1}H NMR ($CDCl_3$, 600 MHz) δ 6.37 (1H, dd, J = 15.1, 10.4 Hz, H-7), 6.17 (1H, dd, J = 15.1, 10.7 Hz, H-14), 6.01 (1H, dd, J = 15.3, 10.4 Hz, H-8), 5.80 (1H, dd, J = 15.3, 7.2 Hz, H-9), 5.76 (1H, d, J = 10.8 Hz, H-13), 5.48 (2H, td, J = 15.8, 7.8 Hz, H-6, H-15), 4.94 (1H, d, J = 7.8 Hz, H-5), 4.57 (1H, s, H-2), 3.52 (1H, s, H-3), 2.44 (1H, m, H-10), 2.11 (2H, m, H_a-11, H-16), 1.98 (1H, m, H_b-11), 1.71 (3H, s, CH_3-12), 1.47 (3H, s, CH_3-4), 1.32 (2H, m, H-17), 0.99 (6H, t, J = 7.2 Hz, CH_3-10, CH_3-16), 0.86 (3H, t, J = 7.4 Hz, H-18); ^{13}C NMR ($CDCl_3$, 150 MHz) δ 170.7 (C-1), 145.4 (C-9), 139.1 (C-15), 138.1 (C-7), 133.8 (C-12), 127.2 (C-13), 126.2 (C-8), 124.8 (C-14), 122.2 (C-6), 80.4 (C-4), 68.2 (C-2), 58.7 (C-3), 58.4 (C-4), 47.4 (C-11), 38.8 (C-16), 35.0 (C-10), 30.0 (C-17), 20.3 (CH_3-16), 19.6(CH_3-10), 18.0(CH_3-4), 16.7(CH_3-12), 11.9 (C-18).

Compound **5**: Pale yellow oil; ^{1}H NMR (CD_3OD, 600 MHz) δ 6.71 – 6.65 (2H, m, H-5, H-2), 6.56 (1H, dd, J = 8.0, 2.1 Hz, H-6), 3.66 (3H, s, H-OMe), 3.46 (2H, s, H-7); ^{13}C NMR (Methanol-d_4, 150 MHz) δ 174.6 (C-8), 146.3 (C-3), 145.5 (C-4), 126.9 (C-1), 121.6 (C-6), 117.3 (C-2), 116.3 (C-5), 52.4 (C-OMe), 41.2 (C-7).

Compound **6**：Pale yellow oily；^{1}H NMR (CD_3OD, 600 MHz) δ 7.09 – 7.06 (2H, m,H-4, H-4′), 6.74 – 6.70 (2H, m, H-5, H-5′), 3.66 (3H, s, H-7), 3.53 (2H, s, H-2); ^{13}C NMR (CD_3OD, 150 MHz) δ 174.6 (C-1), 157.6 (C-6), 131.3 (C-5, C-5′), 126.3 (C-3), 116.2 (C-4, C-4′), 52.4 (C-7), 40.9 (C-2).

Compound **7**：White powder；^{1}H NMR ($CDCl_3$, 600 MHz) δ 8.08 (1H, brs, -NH), 7.62 (1H, dd, *J* = 8.0, 1.1 Hz, H-4), 7.37 (1H, dt, *J* = 8.2, 0.9 Hz, H-7), 7.21 (2H, m, H-5, H-2), 7.14 (1H, ddd, *J* = 8.0, 7.0, 1.0 Hz, H-6), 3.79 (2H, d, *J* = 0.9 Hz, H-8), 3.70 (3H, s, -OMe); ^{13}C NMR ($CDCl_3$, 150 MHz) δ 172.6 (C-COO-), 136.2 (C-7a), 127.4 (C-3a), 123.1 (C-2), 122.4 (C-6), 119.9 (C-5), 119.0 (C-4), 111.3 (C-7), 108.7 (C-3), 52.1 (C-OCH_3), 31.3 (C-8).

Compound **8**：White powder；^{1}H NMR ($CDCl_3$, 600 MHz) δ 12.94 (1H, brs, 5-OH), 6.31 (2H, q, *J* = 2.3 Hz, H-6, H-8), 3.84 (3H, s, 7-OMe), 2.37 (3H, s, H-9), 2.00 (3H, s, H-10); ^{13}C NMR ($CDCl_3$, 150 MHz) δ 182.1 (C-4), 165.2 (C-7), 162.6 (C-2), 162.1 (C-5), 157.7 (C-8a), 115.2 (C-3), 104.8 (C-4a), 97.8 (C-6), 91.9 (C-8), 55.8 (7-OMe), 18.6 (C-9), 9.3 (C-10).

Compound **9**：Pale green crystal；^{1}H NMR (CD_3OD, 600 MHz) δ 6.46 (1H, d, *J* = 2.2 Hz, H-8), 6.30 (1H, d, *J* = 2.2 Hz, H-6), 4.54 (2H, brs, H-10), 3.86 (3H, s, 7-OMe), 2.49 (3H, s, H-9); ^{13}C NMR (CD_3OD, 150 MHz) δ 182.6 (C-4), 168.5 (C-7), 167.2 (C-2), 163.2 (C-5), 159.2 (C-8a), 119.6 (C-3), 105.8 (C-4a), 98.9 (C-6), 93.1 (C-8), 56.4 (C-10), 54.5 (C-OMe), 18.1 (C-9).

Compound **10**: White powder; ^{1}H NMR (CD_3OD, 600 MHz) δ 5.98 (1H, s, H-5), 2.19 (3H, s, 6-Me), 1.84 (3H, s, 3-Me); ^{13}C NMR (CD_3OD, 150 MHz) δ 169.2 (C-2), 168.9 (C-4), 161.3 (C-6), 102.1 (C-5), 98.5 (C-3), 19.5 (6-Me), 8.2 (3-Me).

Compound **11**: White powder; ^{1}H NMR (CD_3OD, 600 MHz) δ 8.55 (1H, s, H-3′), 7.29 (1H, d, J = 8.7 Hz, H-5′), 7.08 (1H, d, J = 8.7 Hz, H-6′), 5.71 (1H, ddd, J = 10.4, 2.1 Hz, H-7), 5.64 (1H, ddd, J = 10.4, 2.3 Hz, H-6), 4.91 (1H, q, J = 2.5 Hz, H-5), 4.24 (1H, dq, J = 7.8, 2.5 Hz, H-9), 4.19 (1H, dd, J = 8.1, 2.3 Hz, H-8), 3.95 (3H, s, 10′-Me), 3.92 (3H, s, 11′-Me), 2.82 (1H, dd, J = 19.2, 2.4 Hz, H-3a), 2.32 (1H, d, J = 19.2 Hz, H-3b); ^{13}C NMR (CD_3OD, 150 MHz) δ 162.5 (C-1), 159.7 (C-1′), 155.8 (C-7′), 151.1 (C-2), 145.4 (C-9′), 137.3 (C-8′), 130.4 (C-6), 128.9 (C-7), 125.5 ((C-3′)), 124.0 (C-5′), 122.3 (C-2′), 115.4 (C-4′), 111.1 (C-6′), 85.5 (C-9), 68.9 (C-4), 67.6 (C-5), 66.4 (C-8), 61.8 (C-11′), 57.0 (C-10′), 25.9 (C-3); Crystal Data for $C_{40}H_{40}Cl_2N_4O_{17}$ (M = 919.66 g/mol): monoclinic, space group $P2_1$ (no. 4), a = 13.38463(5) Å, b = 10.91654(7) Å, c = 13.69527(6) Å, β = 91.5372(4) , V = 2000.349(17) Å^3, Z = 2, T = 99.9(2) K, μ(Cu Kα) = 2.196 mm^{-1}, $Dcalc$ = 1.527 g/cm^3, 37482 reflections measured (6.456° ≤ 2Θ ≤ 148.838°), 7501 unique (R_{int} = 0.0288, R_{sigma} = 0.0197) which were used in all calculations. The final R_1 was 0.0304 (I > 2σ(I)) and wR_2 was 0.0824.

Compound **12**: White powder; ^{1}H NMR (CD_3OD, 600 MHz) δ 7.01 (1H, d, J = 8.8 Hz, H-5′), 6.94 (1H, s, H-3′), 6.60 (1H, d, J = 8.9 Hz, H-6′), 6.06 (1H, dd, J = 10.0, 4.8 Hz, H-7), 5.92 (1H, dd, J = 10.1, 5.0 Hz, H-6), 4.34 (1H, dd, J = 5.1, 2.1 Hz, H-8), 4.20 (1H, d, J = 2.2 Hz, H-9), 4.13 (1H, d, J = 5.0 Hz, H-5), 3.87 (3H, s,

7′-OMe), 3.81 (3H, s, 8′-OMe), 3.12 (1H, d, *J* = 11.6 Hz, H_a-3), 2.39 (1H, d, *J* = 11.6 Hz, H_b-3); ^{13}C NMR (CD_3OD, 150 MHz) *δ* 162.1 (C-1), 158.1 (C-1′), 156.0 (C-7′), 149.7 (C-9′), 138.1 (C-8′), 129.5 (C-5′), 128.3 (C-7), 126.1 (C-6), 121.2 (C-2′), 118.8 (C-7), 115.6 (C-4′), 105.5 (C-6′), 81.8 (C-9), 77.2 (C-2), 76.2 (C-4), 65.3 (C-8), 61.1 (8′-OMe), 56.4 (7′-OMe), 50.9 (C-3), 47.7 (C-5); HRESIMS *m*/*z* 447.0871 $[M-H]^-$ (calculated for $C_{20}H_{19}N_2O_8S$, 447.0868).

Compound **13**: Yellow powder; ^1H NMR (DMSO, 600 MHz) *δ* 9.33 (1H, s, H-10′), 8.54 (1H, s, H-3′), 7.50 (1H, d, *J* = 8.8 Hz, H-5′), 7.14 (1H, d, *J* = 8.8 Hz, H-6′), 5.81 (1H, d, *J* = 9.9 Hz, H-5), 5.69 (1H, dd, *J* = 9.9, 5.0 Hz, H-6), 5.52 (1H, s, 4-OH), 4.24 (1H, dd, *J* = 10.9, 1.8 Hz, H-9), 4.05 (1H, dd, *J* = 4.7 Hz, H-7), 3.90 (3H, s, H-11′), 3.84 (3H, s, H-12′), 3.48 (1H, dd, *J* = 11.0, 4.3 Hz, H-8), 2.61 (1H, dd, *J* = 19.1, 2.0 Hz, H_a-3), 2.18 (1H, d, *J* = 18.9 Hz, H_b-3); ^{13}C NMR (DMSO, 150 MHz) *δ* 160.8 (C-1), 157.9 (C-1′), 153.9 (C-7′), 147.7 (C-2), 143.6 (C-9′), 135.6 (C-5), 135.2 (C-8′), 126.8 (C-6), 124.0 (C-3′), 123.1 (C-5′), 120.9 (C-2′), 113.7 (C-4′), 110.1 (C-6′), 79.4 (C-9), 67.7 (C-8), 66.6 (C-7), 64.1 (C-4), 60.9 (C-12′), 56.4 (C-11′), 28.9 (C-3).

Compound **14**: Yellow powder; ^1H NMR (DMSO, 600 MHz) *δ* 9.33 (1H, s, NH), 8.54 (2H, s, H-3′), 7.51 (H, dd, *J* = 8.9, 1.2 Hz, H-5′), 7.14 (H, d, *J* = 8.8 Hz, H-6′), 5.45 (1H, d, H-6), 5.40 (1H, d, *J* = 10.4, 1.9 Hz, H-7), 5.36 (1H, d, 4-OH), 4.23 (H, brd, *J* = 3.0 Hz, H-5), 4.00 (2H, d, *J* = 1.9 Hz, H-9, H-8), 3.90 (3H, s, 10′-Me), 3.84 (3H, s, 11′-Me), 2.48 (1H, dd, *J* =19.2, 1.8 Hz, H_a-3), 2.04 (1H, d, *J* = 19.2 Hz, H_b-3); ^{13}C NMR (DMSO, 150 MHz) *δ* 161.0 (C-1), 157.9 (C-1′), 153.8 (C-7′),

150.2 (C-2), 143.6 (C-9′), 135.2 (C-8′), 129.9 (C-6), 128.1 (C-7), 123.7 (C-3′), 123.1 (C-5′), 121.0 (C-2′), 113.7 (C-4′), 110.1 (C-6′), 83.9 (C-9), 67.4 (C-4), 66.2 (C-5), 63.1 (C-8), 60.9 (C-11′), 56.4 (C-10′), 23.1 (C-3).

Compound **15**：White powder；^{1}H NMR (CD_3OD, 600 MHz) δ 7.55 (1H, dt, *J* = 7.9, 1.0 Hz, H-4), 7.32 (1H, dt, *J* = 8.2, 1.0 Hz, H-7), 7.11 – 7.05 (2H, m, H-5, H-2), 7.00 (1H, ddd, *J* = 8.0, 7.0, 1.0 Hz, H-6), 3.46 (2H, t, *J* = 7.4 Hz, H-10), 2.94 (2H, td, *J* = 7.4, 0.9 Hz, H-11), 1.91 (3H, s, H-14); ^{13}C NMR (CD_3OD, 150 MHz) δ 173.3 (C-13), 138.2 (C-8), 128.8 (C-9), 123.4 (C-6), 122.3 (C-2), 119.6 (C-5), 119.2 (C-4), 113.3 (C-3), 112.2 (C-7), 41.6 (C-11), 26.2 (C-10), 22.6 (C-14).

Compound **16**：Pale yellow powder；^{1}H NMR (CD_3OD, 600 MHz) δ 9.89 (1H, s, CHO), 8.20 – 8.14 (1H, m, H-4), 8.10 (1H, s, H-2), 7.51 – 7.45 (1H, m, H-7), 7.30 – 7.21 (2H, m, H-5, H-6); ^{13}C NMR (CD_3OD, 150 MHz) δ 187.4 (3-CHO), 139.7 (C-2), 138.9 (C-9), 125.7 (C-8), 125.0 (C-6), 123.6 (C-4), 122.4 (C-5), 120.1 (C-3), 113.1 (C-7).

Compound **17**：White powder；^{1}H NMR (CD_3OD, 600 MHz) δ 6.94 (1H, dd, *J* = 2.5, 1.5 Hz, H-4), 6.86 (1H, dd, *J* = 3.7, 1.5 Hz, H-2), 6.18 (1H, dd, *J* = 3.7, 2.5 Hz, H-3); ^{13}C NMR (CD_3OD, 150 MHz) δ 164.5 (C-6), 124.4 (C-2), 123.9 (C-5), 116.6 (C-4), 110.6 (C-3).

Compound **18**：Colorless oily；^{1}H NMR ($CDCl_3$, 600 MHz) δ 5.12 (1H, tp, *J* = 7.3, 1.5 Hz, H-9), 2.04 (2H, m, H-8), 1.85 (2H, m, H-4, H_a-5), 1.68 (4H, m, H-11, H_b-5), 1.62 (4H, m, H-15, H_a-6), 1.58 (1H, m, H_b-6), 1.48 (2H, t, *J* = 8.3 Hz, H-7), 1.25 (3H, s, H-12), 1.16 (3H, s, H-14), 1.04 (3H, d, *J* = 6.9 Hz, H-14); ^{13}C NMR

($CDCl_3$, 150 MHz) δ 131.9 (C-10), 124.6 (C-9), 81.5 (C-1), 75.0 (C-3), 54.4 (C-4), 44.4 (C-2), 40.5 (C-6), 40.5 (C-7), 26.2 (C-12), 25.9 (C-14), 25.2 (C-11), 24.5 (C-5), 22.8 (C-8), 17.8 (C-15), 14.7 (C-13).

Compound **19**: Yellow powder; ^{1}H NMR (CD_3OD, 600 MHz) δ 7.59 (1H, d, *J* = 15.9 Hz, H-β), 7.18 (1H, d, *J* = 1.9 Hz, H-2), 7.06 (1H, dd, *J* = 8.2, 2.0 Hz, H-6), 6.81 (1H, d, *J* = 8.2 Hz, H-5), 6.31 (1H, d, *J* = 15.9 Hz, H-α), 3.89 (3H, s, -OMe); ^{13}C NMR (CD_3OD, 150 MHz) δ 171.0 (CO), 150.5 (C-3), 149.4 (C-4), 146.9 (C-β), 127.8 (C-1), 124.0 (C-6), 116.5 (C-5), 116.0 (C-α), 111.7 (C-2), 56.4 (-OMe).

Compound **20**: Yellow powder; ^{1}H NMR ($CDCl_3$, 600 MHz) δ 7.33 (2H, m, -Ph), 7.28 (3H, m, -Ph), 3.64 (2H, s, H-2); ^{13}C NMR ($CDCl_3$, 150 MHz) δ 177.5 (C-1), 133.5 (C-1′), 129.5 (C-2′, C-6′), 128.8 (C-3′,C-5′), 127.5 (C-4′), 41.3 (C-2).

Compound **21**: Colorless oily; ^{1}H NMR ($CDCl_3$, 600 MHz) δ 7.14 (2H, d, *J* = 8.4 Hz, H-2, H-6), 6.77 (2H, d, *J* = 8.5 Hz, H-3, H-5), 3.69 (3H, s, -OMe), 3.56 (2H, s, H-7); ^{13}C NMR ($CDCl_3$, 150 MHz) δ 172.7 (C-8), 154.9 (C-4), 130.6 (C-1), 126.2 (C-2, C-6), 115.6 (C-3, C-5), 52.2 (-OMe), 40.4 (C-7).

Compound **22**: Brown crystals; ^{1}H NMR (CD_3OD, 600 MHz) δ 7.08 (2H, m, H-2, H-6), 6.72 (2H, m, H-3, H-5), 3.48 (2H, s); ^{13}C NMR (CD_3OD, 150 MHz) δ 176.3 (C-8), 157.4 (C-4), 131.3 (C-2, C-6), 126.8 (C-1), 116.2 (C-3, C-5), 41.1 (C-7).

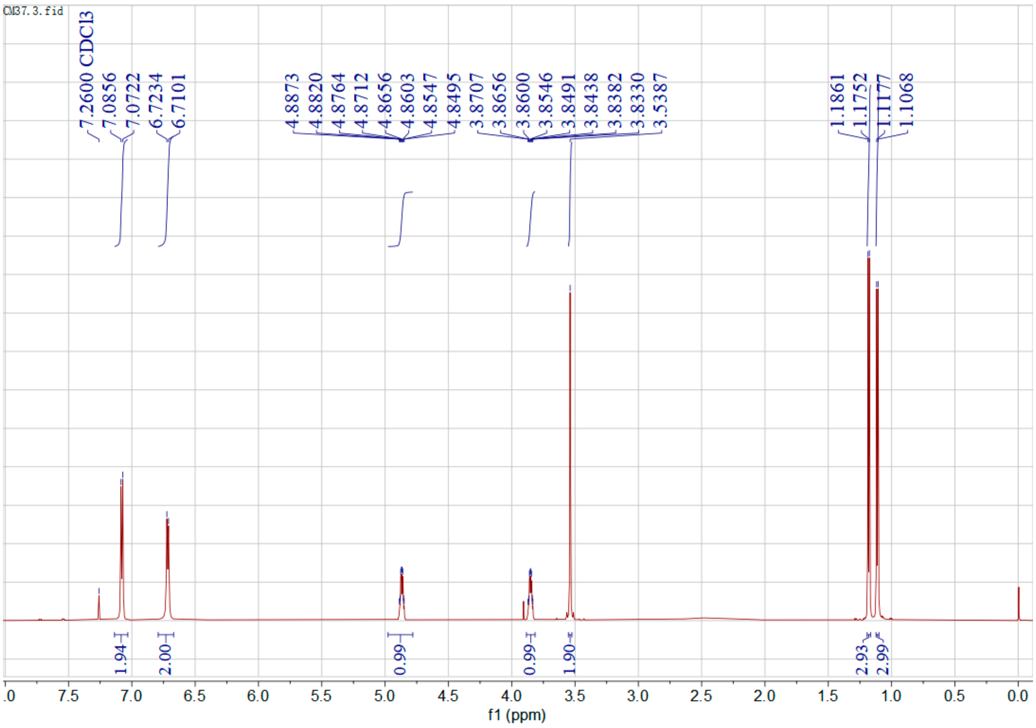

Figure S1. ^{1}H NMR spectrum of (±)-penilactone F (**1**) in $CDCl_3$

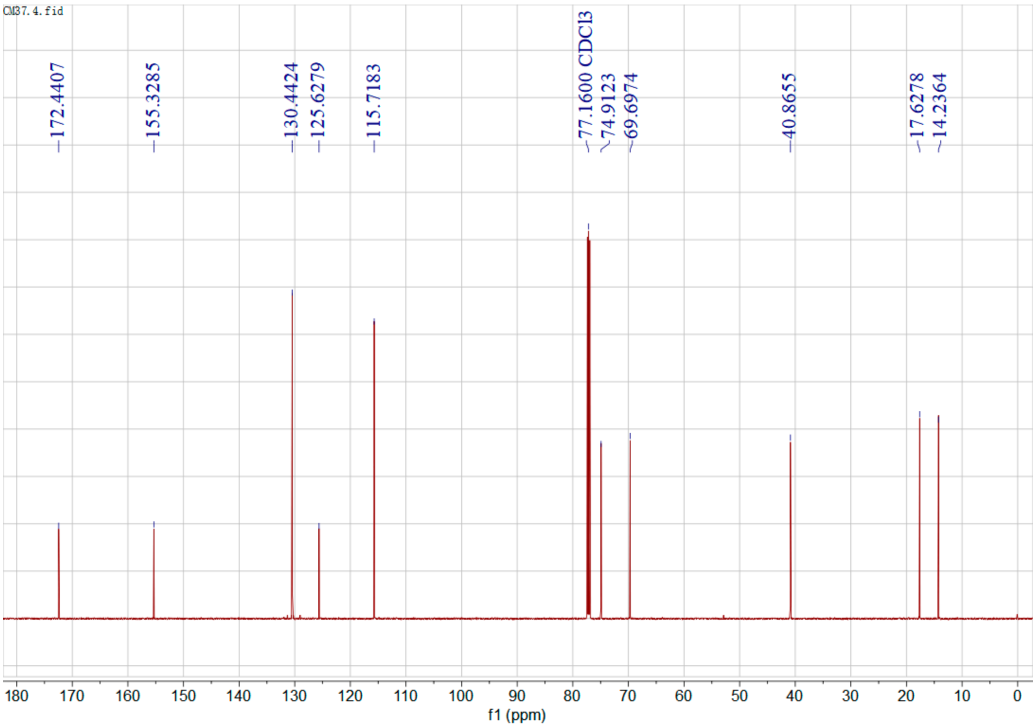

Figure S2. ^{13}C NMR spectrum of (±)-penilactone F (**1**) in $CDCl_3$

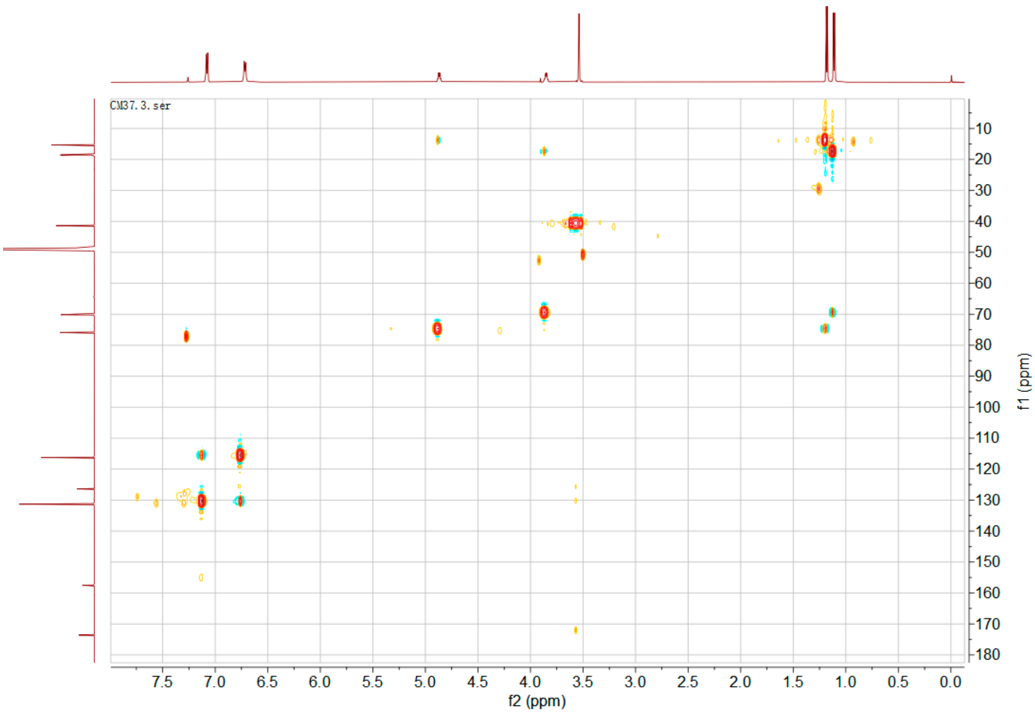

Figure S3. HSQC spectrum of (±)-penilactone F (**1**) in $CDCl_3$

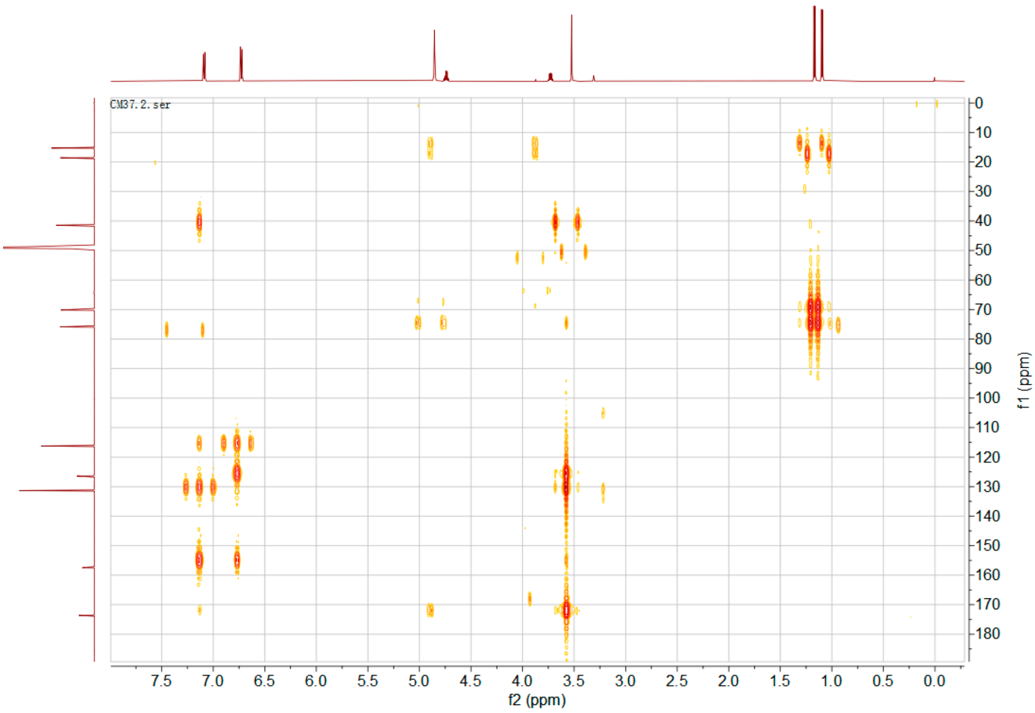

Figure S4. HMBC spectrum of (±)-penilactone F (**1**) in $CDCl_3$

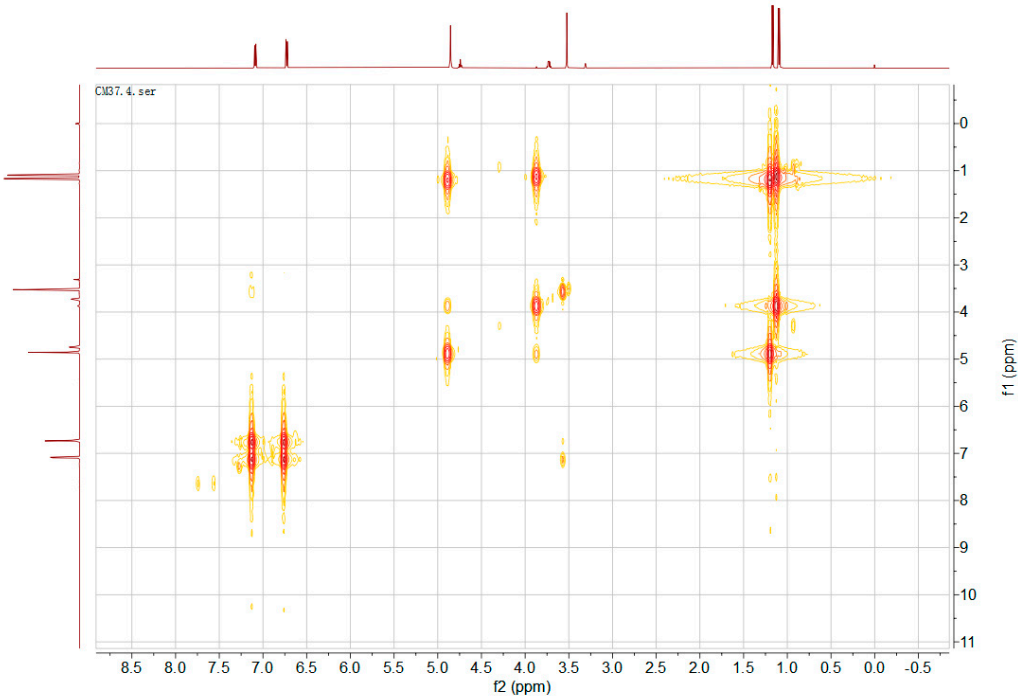

Figure S5. ^{1}H-^{1}H COSY spectrum of (±)-penilactone F (**1**) in $CDCl_3$

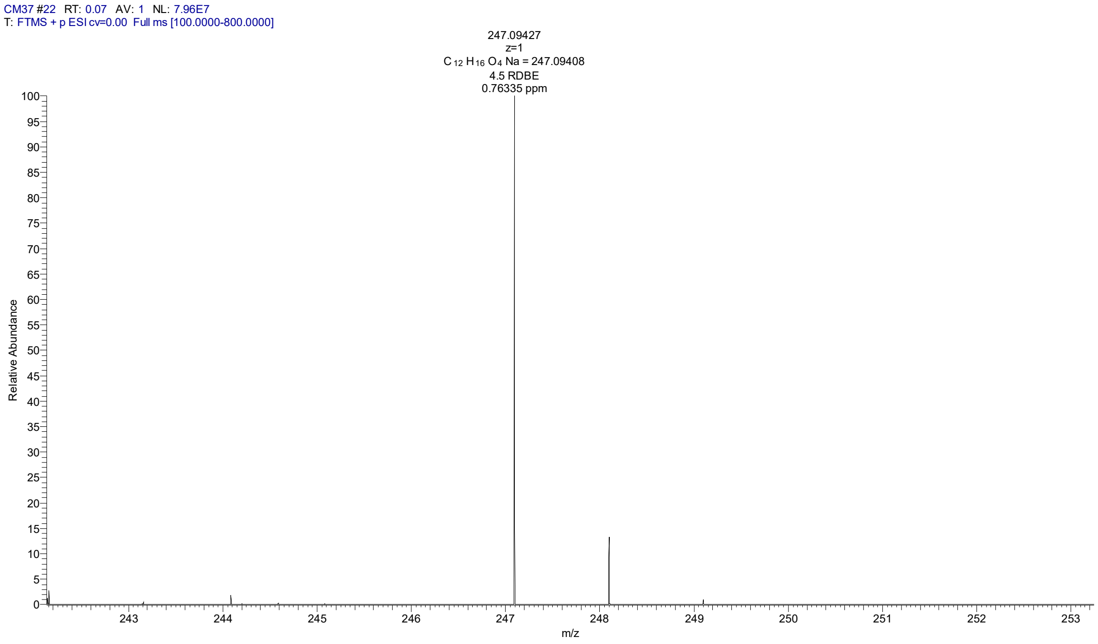

Figure S6. HRESIMS spectrum of (±)-penilactone F (**1**)

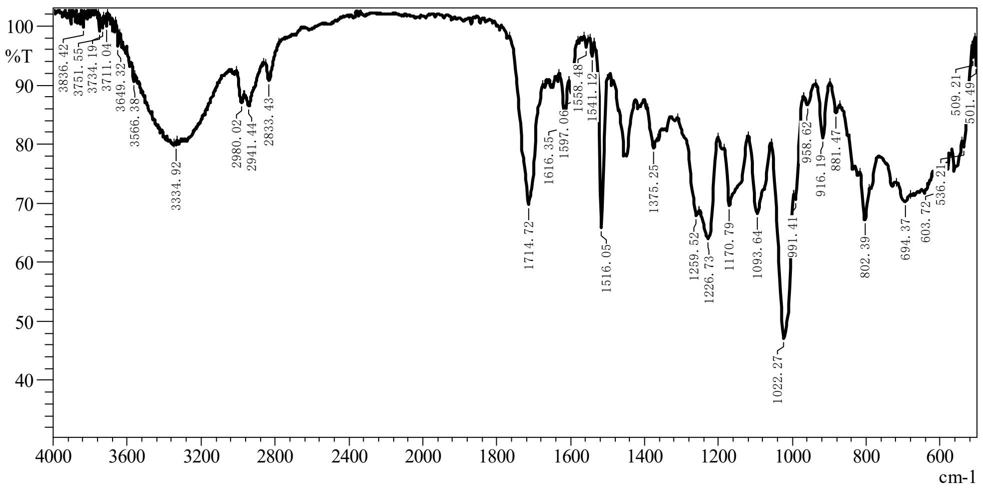

Figure S7. IR spectrum of (±)-penilactone F (**1**)

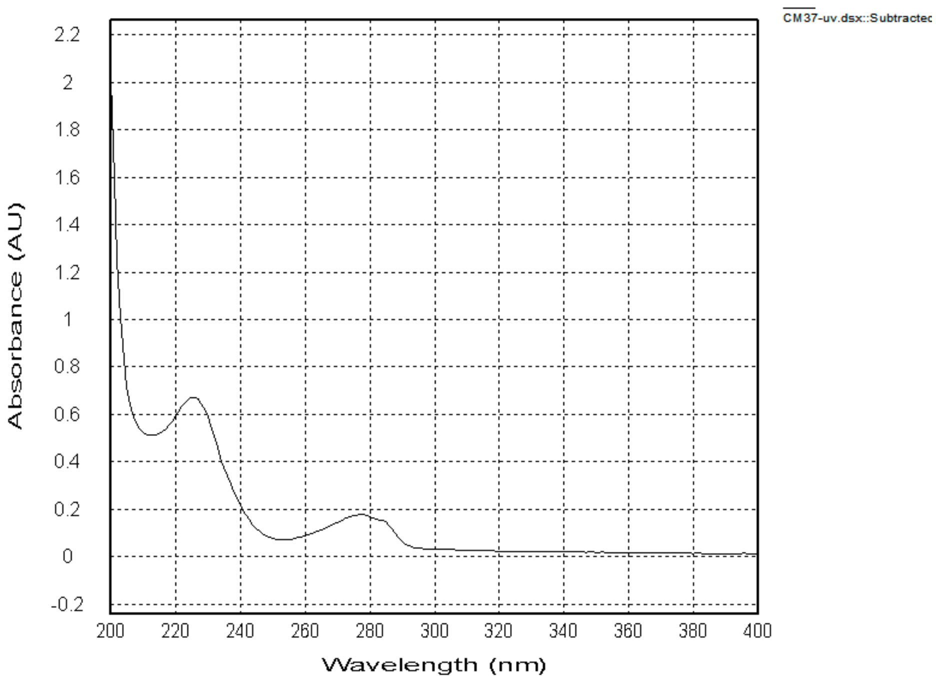

Figure S8.UV spectrum of (±)-penilactone F (**1**) in MeOH

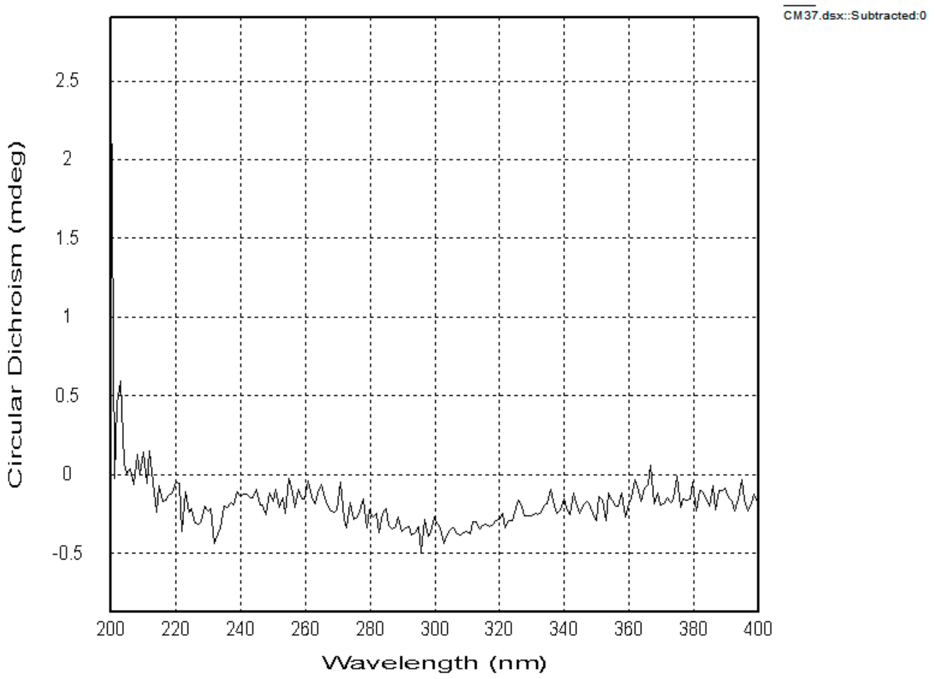

Figure S9. ECD spectrum of (±)-penilactone F (**1**) in MeOH

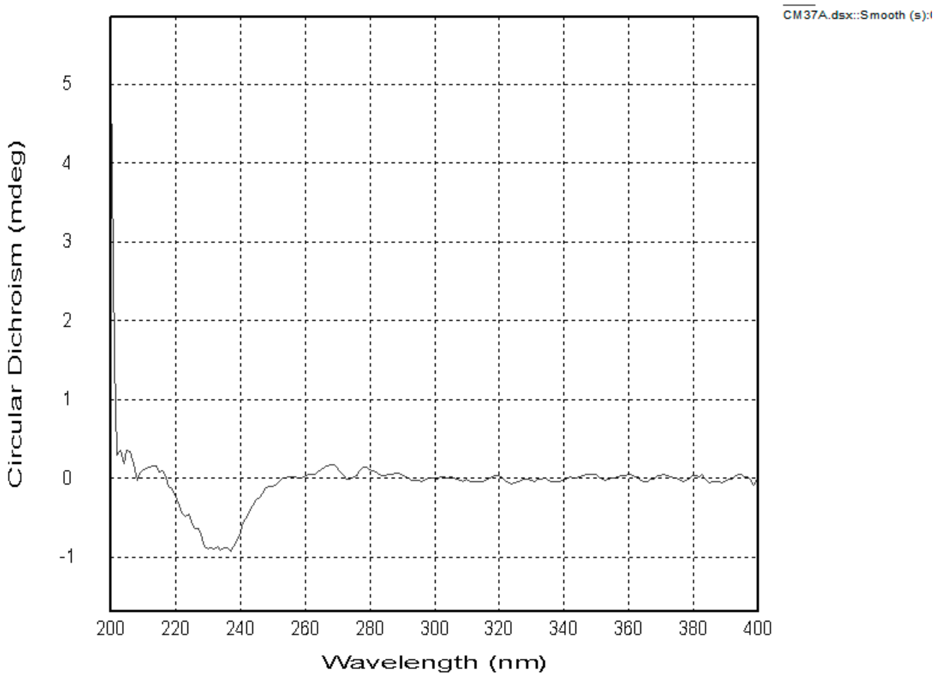

Figure S10. ECD spectrum of (+)-Penilactone F((+) -**1**) in MeOH

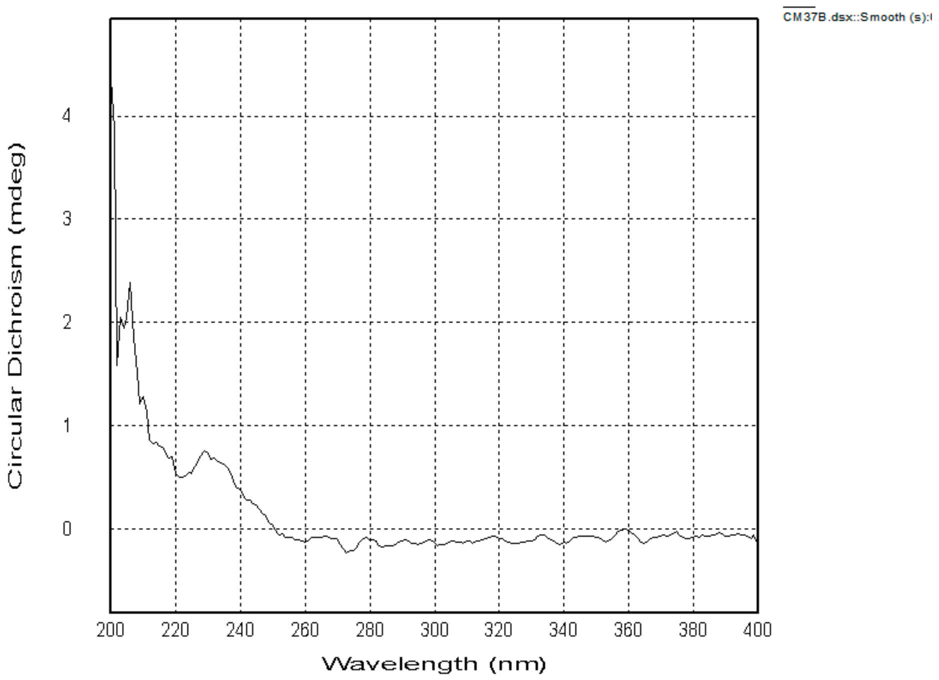

Figure S11. ECD spectrum of (−)-Penilactone F((-) -**1**) in MeOH

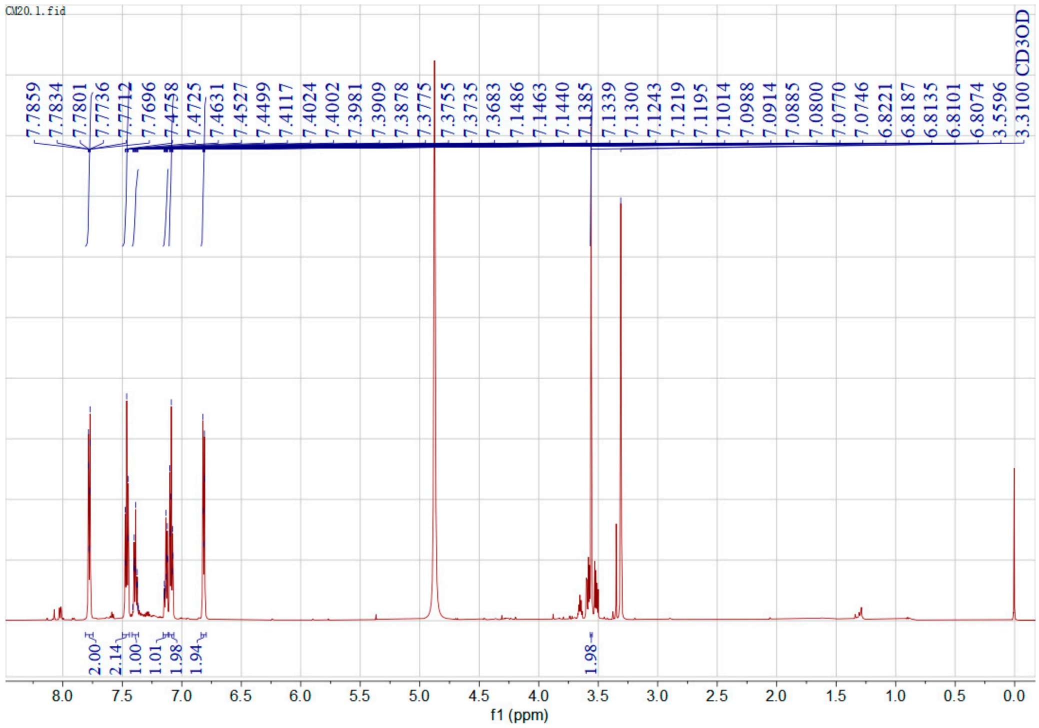

Figure S12. ^{1}H NMR spectrum of (±) phenylbutyrolactone IIa (**2**) in CD_3OD

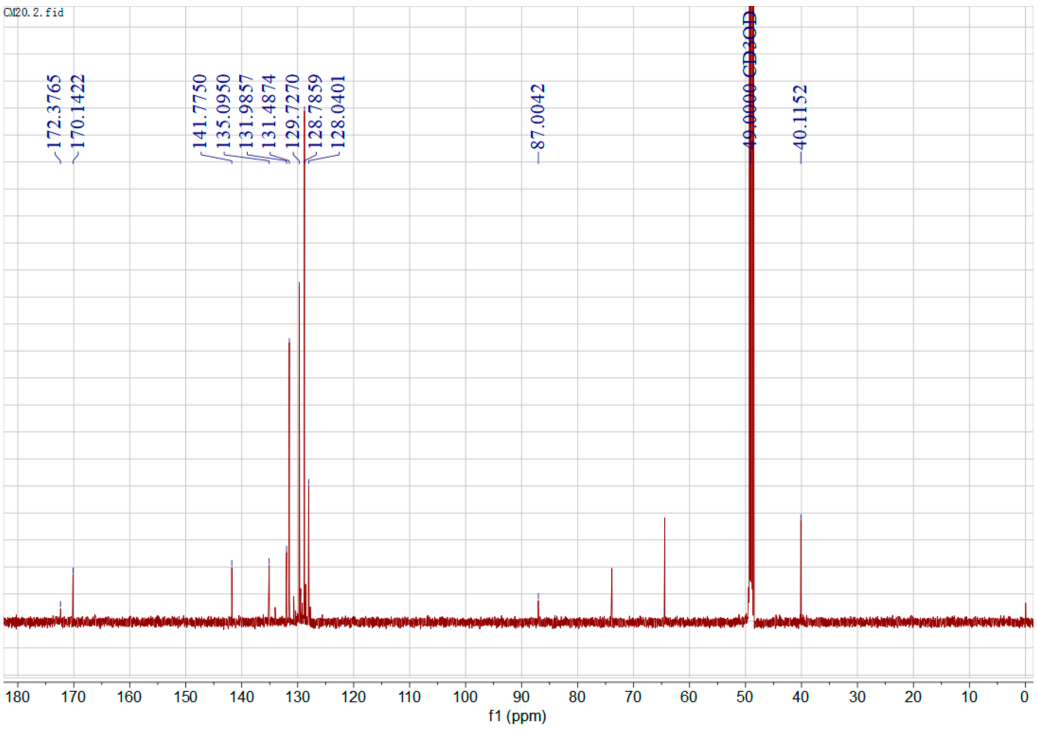

Figure S13. ^{13}C NMR spectrum of (±) phenylbutyrolactone IIa (**2**) in CD_3OD

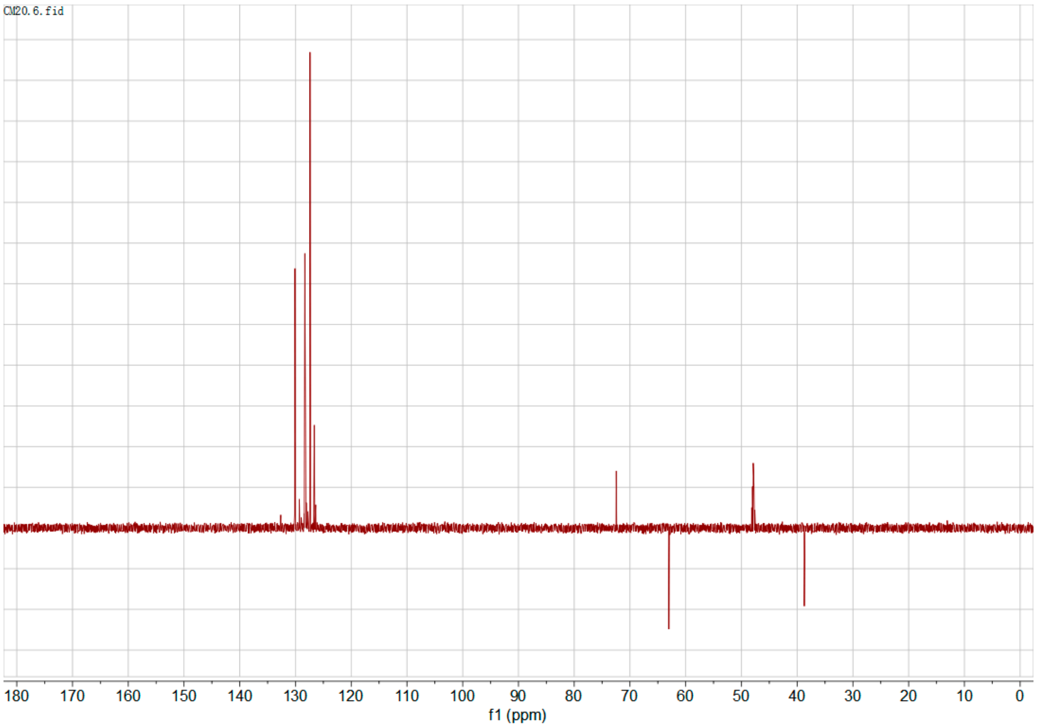

Figure S14. DEPT135 spectrum of (±) phenylbutyrolactone IIa (**2**) in CD_3OD

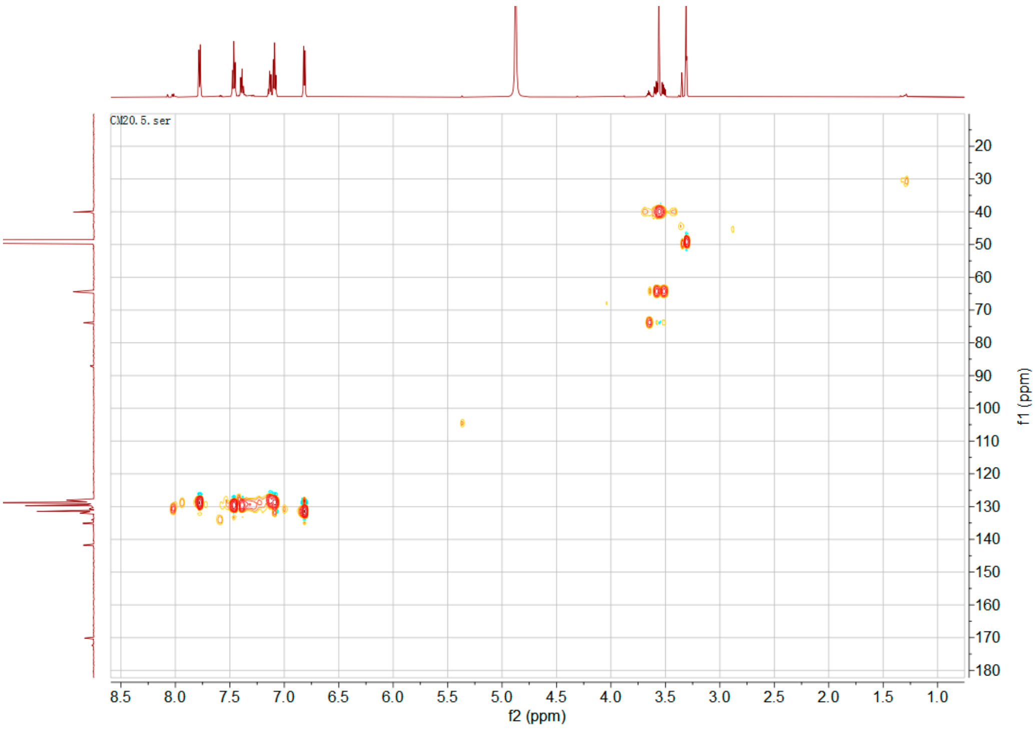

Figure S15. HSQC spectrum of (±) phenylbutyrolactone IIa (**2**) in CD_3OD

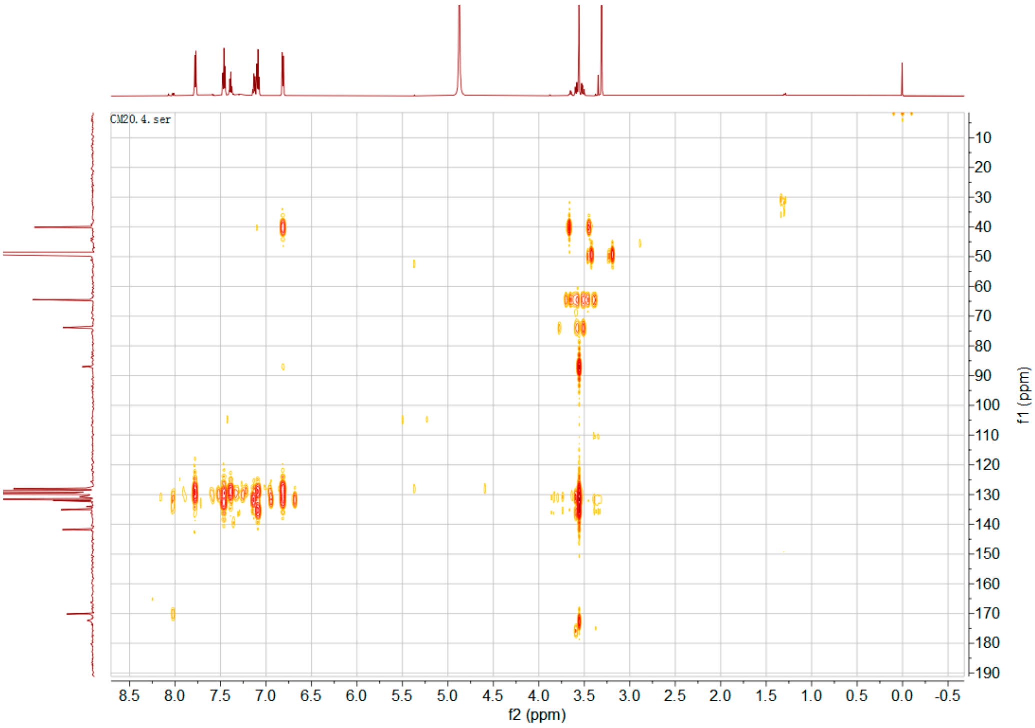

Figure S16. HMBC spectrum of (±) phenylbutyrolactone IIa (**2**) in CD_3OD

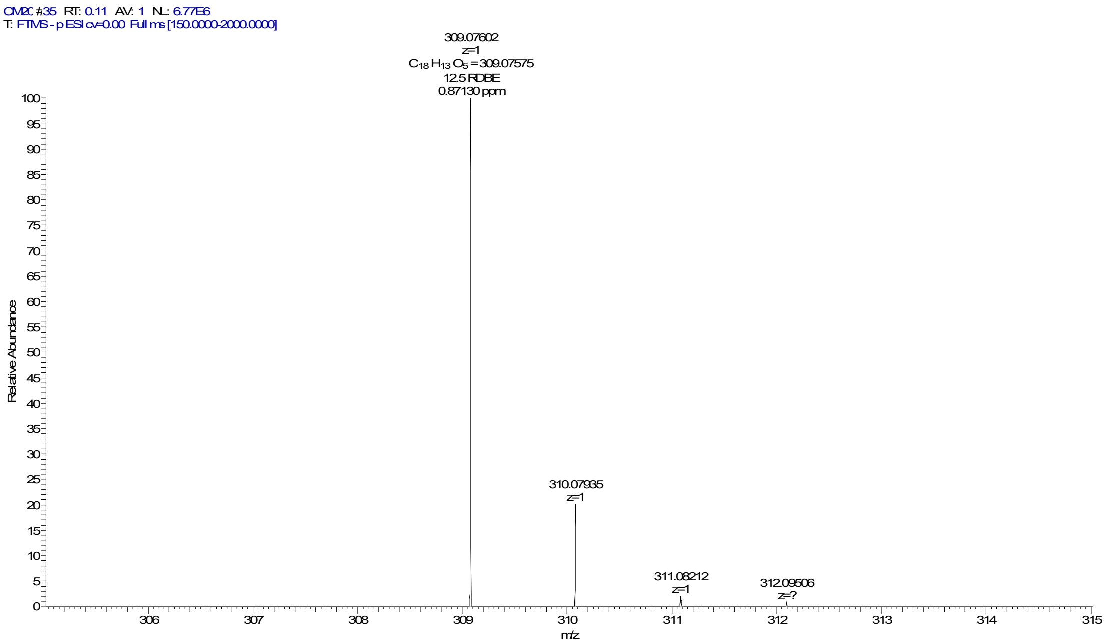

Figure S17. HRESIMS spectrum of (±) phenylbutyrolactone IIa (**2**)

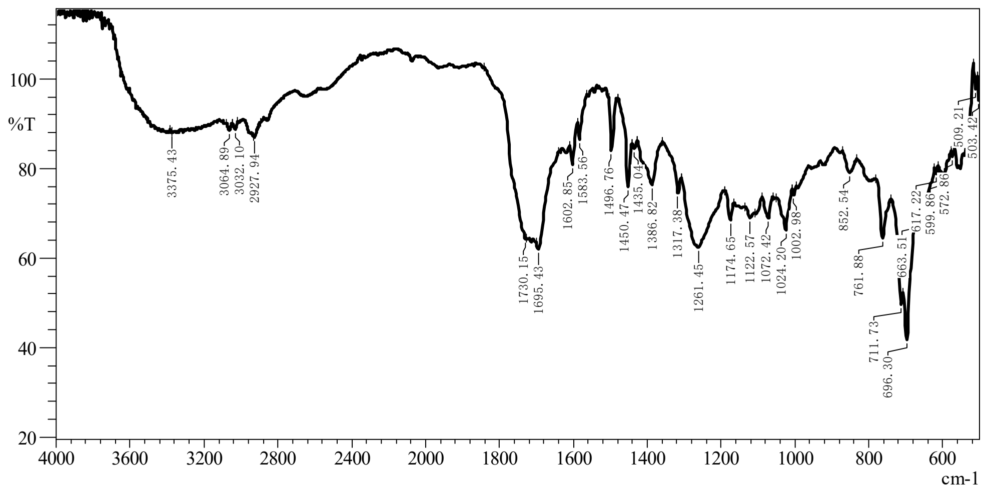

Figure S18. IR spectrum of (±) phenylbutyrolactone IIa (**2**)

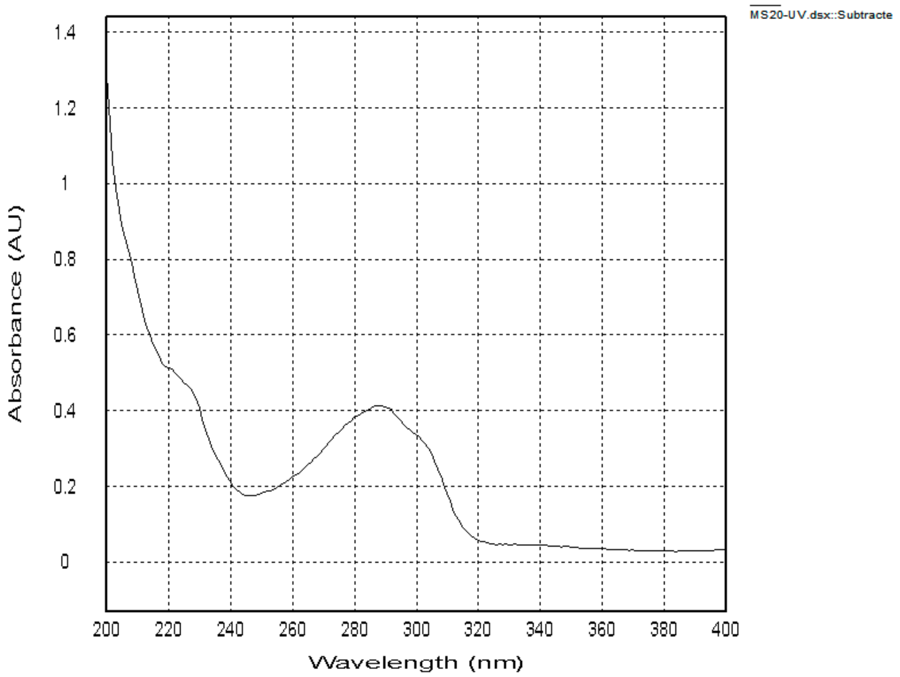

Figure S19.UV spectrum of (±) phenylbutyrolactone IIa (**2**) in MeOH

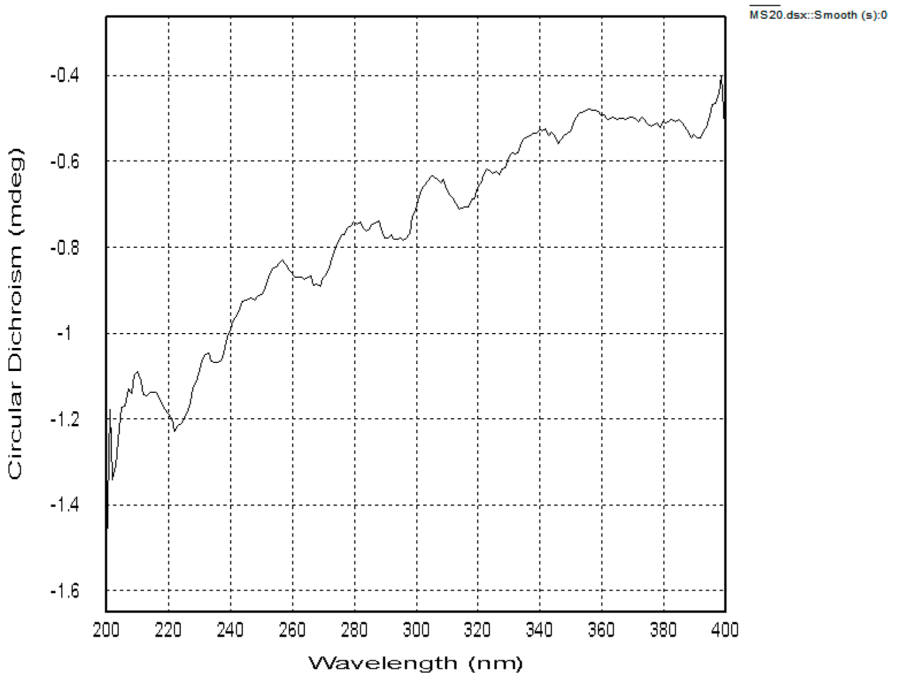

Figure S20. ECD spectrum of (±) phenylbutyrolactone IIa (**2**) in MeOH

The strain's (*Aspergillus spelaeus* SCSIO 41433) ITS sequence of the rDNA

GTAACAAGGTTTCCGTAGGTGAACCTGCGGAAGGATCATTACCGAGTG

AGGGTCCTCGTGGCCCAACCTCCCACCCGTGACTATTGTACCTTTGTTG

CTTCGGCGGGCCCGCCAGCCCCGTGCTGGCCGCCGGGGGGCCTTGTGC

CCCCGGGTCCGCGCCCGCCGGAGACCCCAACATGAACACTGTTCTGA

AAGCCTGTATGAATCCGATTCTTTGTAATCAGTTAAAACTTTCAACAAT

GGATCTCTTGGTTCCGGCATCGATGAAGAACGCAGCGAAATGCGATAA

CTAATGTGAATTGCAGAATTCAGTGAATCATCGAGTCTTTGAACGCACA

TTGCGCCCCCTGGTATTCCGGGGGGCATGCCTGTCCGAGCGTCATTGCT

GCCCTCAAGCCCGGCTTGTGTGTTGGGTCCTCGTCCTCCCCTCGCGGGG

GGACGGGCCCGAAAGGCAGCGGCGGCACCGCGTCCGGTCCTCGAGCG

TATGGGGCTTTGTCACCCGCTCTGTAGGCCCGGCCGGCGCCAGCCGAC

GCAAATCACCTTTTTTTTTCAGGTTGACCTCGGATCAGGTAGGATACC

CGCTGAACTTAAGCA
